# The Clonic Phase of Seizures in Patients Treated with Electroconvulsive Therapy is Related to Age and Stimulus Intensity

**DOI:** 10.3389/fpsyt.2013.00166

**Published:** 2013-12-23

**Authors:** Chao-Chih Wang, Ching-Hung Lin, Yao-Chu Chiu, Chih-Chieh Tseng

**Affiliations:** ^1^Department of Psychology, National Chung Cheng University, Chiayi, Taiwan; ^2^Department of Psychology, Soochow University, Taipei, Taiwan; ^3^Biomedical Engineering R&D Center, China Medical University Hospital, Taichung, Taiwan; ^4^Biomedical Electronics Translational Research Center, National Chiao Tung University, Hsinchu, Taiwan; ^5^Department of Psychology, Kaohsiung Medical University, Kaohsiung, Taiwan; ^6^Department of Psychiatry, Beitou Branch, Tri-Service General Hospital, National Defense Medical Center, Taipei, Taiwan; ^7^National Taipei University of Nursing and Health Sciences, Taipei, Taiwan

**Keywords:** seizure duration, tonic phase, clonic phase, age, electroconvulsive therapy

## Abstract

**Background**: Electroconvulsive therapy (ECT) is effective in the treatment of major depressive disorder and schizophrenia in patients who are drug-naïve or less-receptive to antipsychotic drugs. Several studies have discussed the correlation between patient characteristics, input-current volume, and seizure duration. According to the present principle of ECT guidelines, the therapeutic effect of ECT mostly correlates with seizure duration. As the tonic phase is different from the clonic phase with respect to brain function and activity, it is informative to analyze both the tonic and clonic phases. Thus, this study sought to clarify the relationship between the features of the two phases, and to re-examine and refine guidelines regarding ECT treatment.

**Method**: ECT-course data from 44 schizophrenia or bipolar I patients were recollected, including the number of treatments that they had received, their gender, age, and the association of these characteristics with motor seizure duration was analyzed. A two-factor correlation was employed to test the relationship between each of the two factors.

**Result**: The post-analysis results indicate that seizure duration and age are significantly correlated. Older patients had relatively short seizure durations after ECT-treatment. Notably, a negative correlation was only found between age and the clonic phase of the seizure, not between age and the tonic phase. Furthermore, this study also found an inverse relationship between ECT-intensity and the clonic phase, but not between ECT-intensity and the tonic phase.

**Conclusion**: This study demonstrated that age and ECT-intensity are negatively correlated with seizure duration, particularly in the clonic phase. The present observations are not fully consistent with the basic guidelines of the APA-ECT practical manual. Accordingly, the predictions regarding the therapeutic effect of ECT can be based on both the seizure duration and the clonic phase.

## Introduction

Research indicates that twenty percent of schizophrenia patients are drug-naïve (1). Furthermore, when antipsychotics fail to control the psychotic symptoms of schizophrenia, electroconvulsive therapy (ECT) is frequently the last-resort treatment for controlling these symptoms by resetting the brain asynchronization. The ECT procedure involves inputting a certain degree of electrical current into the patient’s brain for a few seconds. This current induces convulsions, which means the neuronal circuitries are simultaneously activated by the external electrical stimulation; namely, the neurons in the brain depolarize at the same time. The induced seizure resembles an epileptic seizure: patients experienced the tonic phase first, with a duration of a few seconds, followed by the clonic phase, with a duration of tens of seconds. After ECT treatment, the majority of the patients recover from the symptoms in a few days to a few weeks.

The basic practical guidelines of the American Psychiatric Association for ECT suggested that the therapeutic effect of ECT could be associated with gender, age, and current input intensity. Specifically, male and elderly patients exhibit worse therapeutic effect following ECT treatment, but a high-intensity current can improve the therapeutic effect. Additionally, there are many practitioners who believe that if the generalized seizure duration is less than 15 s, it is difficult to achieve the therapeutic purpose (2). Namely, if the seizure cannot be completely induced and the brain could not be entirely reset, and if the patient has difficulty resuming normal activity, like normal eating or appropriate interaction with medical staff or others, this trial of ECT therapy is considered a malfunctioning trial.

However, there are some questions that must be clarified with respect to the effects of ECT. For example, seizure duration in patients has been frequently investigated. ECT is known to work better for drug-naïve patients, but the mechanism by which it functions is unclear (2). Generally speaking, many studies have suggested that if there is not a sufficient seizure duration, the effects of the treatment may not be observable (1, 2). This does not mean that seizure duration is a unique, stable index of the treatment effect of ECT. Furthermore, some studies have observed that seizure duration does not correlate with clinical efficacy (3). Therefore, it is still unclear how the therapeutic effects influence patients even though the ECT parameter is particularly useful. However, there have been many studies devoted to determining the correlation between personal factors (age, gender, periods, and type of schizophrenia, etc.) and ECT treatment. Notably, seizure duration can be objectively classified into tonic and clonic phases. It is particularly noteworthy to distinguish the clonic phase from the tonic phase as the clonic phase plays a different role than the tonic phase; therefore, this study examined the relationship between seizure stage and age, as well as that with current intensity, in the hope that the study data can provide further detail to clarify the effects of ECT treatment.

## Materials and Methods

The aim of this study was to perform a retrospective chart survey to test the correlation among the stimulation intensity, patient age, and seizure duration (clonic phase or tonic phase). The patient criteria mostly fitted the standard of the Diagnostic and Statistical Manual of Mental Disorders, 5th ed. (DSM-V). The subjects were hospitalized because of serious psychiatric symptoms in the Beitou Armed Forces Hospital and were treated between January 2007 and December 2008 using ECT. The study consisted of 44 cases (19 male and 25 female) with schizophrenia (*N* = 34, 77%) or bipolar I disorder (*N* = 10, 23%). The subjects had been treated at least eight times using ECT over a therapeutic term exceeding 14 days. Those cases that met the preceding requirements were included in the study. Moreover, any patient who was treated by ECT more than two times during the research term, only the condition of the first treatment was recorded. The length of the convulsions in patients receiving ECT was recorded by the doctors who were conducting the treatment. The doctors simply recorded what they saw and differentiated between tonic and clonic phases based on their naked-eye observations. The diagnosis, the age of the subject receiving ECT and the therapeutic power consumption of each patient were included in the data. All patients were anesthetized using Citosol (1–1.5 mg/kg) with no other drugs. The subjects were not allowed to take any drugs for sleep the previous evening, and they were not allowed to consume any food after 00:00 am. The subjects’ blood pressures, heart rates and respiratory times were monitored, and 3–5 L/min of 100% oxygen was administered by a nasal oxygen cannula at all times during the therapeutic process. All cases were treated by bitemporal electrode placement. All patients remained in the recovery room until their physical conditions were stable. They were then returned to their rooms. This study is a post-survey study and these data are retrieved from the database of Beitou Armed Forces Hospital. All data were collected, analyzed, and reported anonymously.

### Statistics and analysis

The statistical package for SPSS 12.0 was employed for the correlation test and the two-sample *t-*test. The Pearson correlation coefficient was utilized to test the correlation between each of the factors, including stimulus intensity, age, and seizure duration (tonic and clonic phases). The independent *t-*test was used to test gender differences.

## Results

**Figure 1:**
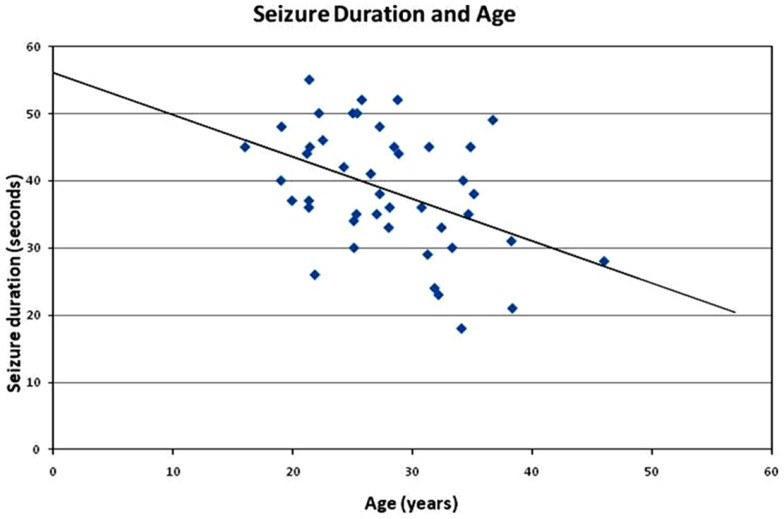
**Correlation of seizure duration with age**. The seizure duration is negatively correlated with patient age (*r* = −0.44, *p* < 0.01). The present finding identified a relationship between the two factors in previous ECT studies and clinical observations.

**Figure 2:**
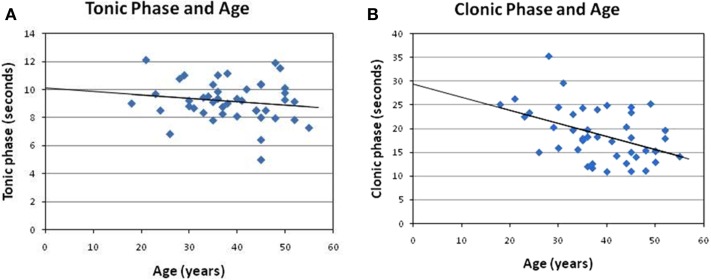
**Correlation of the clonic phase and tonic phases with age**. In the present analysis, **(A)** no significant correlation can be observed between the tonic phase and age (*r* = −0.16, *p* = 0.31). **(B)** However, a negative correlation between clonic duration and age was found here (*r* = −0.455, *p* < 0.01).

**Figure 3:**
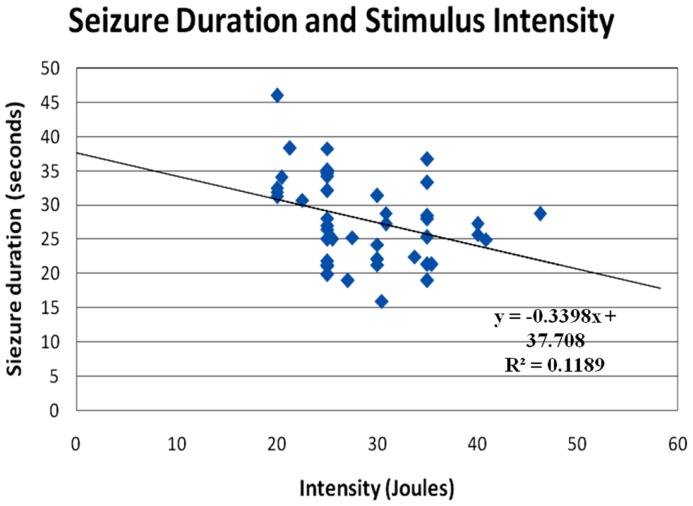
**Correlation of seizure duration with stimulus intensity**. The seizure duration was negatively correlated to the stimulus intensity (*r* = −0.345, *p* < 0.05). However, the present observation may indicate that patients in whom it was difficult to induce seizures required significantly more power input to activate the neuronal circuitry in the brain.

**Figure 4:**
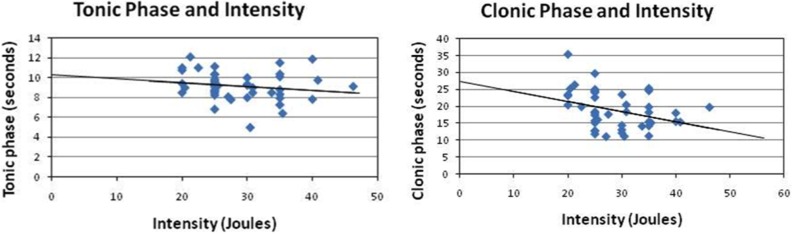
**Correlation of stimulus intensity with the clonic phase and the tonic phase**. While there was an insignificant correlation between the tonic phase and the input power (*r* = −0.17, *p* = 0.27), there was a negative correlation between seizure duration and the input power (*r* = −0.35, *p* < 0.05). The present study demonstrated that the clonic phase might serve as an index for measuring the integrity of neuronal circuitry in patients. Thus, serious brain degeneration requires more power input to trigger a self-induced seizure.

The post-analysis result of the independent *t*-test indicated that there was no statistically significant difference between male and female patients with respect to the stimulus intensity or seizure duration during either the tonic phase or the clonic phase. The data are presented in Tables 1 and 2. There was a significant correlation between age and seizure duration (*r* = −0.435, *p* < 0.01) (Figure 1). Furthermore, the analysis indicated that there was a significant correlation between age and the duration of the clonic phase (*r* = −0.455, *p* < 0.01), but not that of the tonic phase (Figure 2). There was a statistically correlation between stimulus intensity and seizure duration (*r* = −0.345, *p* < 0.05) (Figure 3). Moreover, the additional analysis indicated that there was a significant correlation between stimulus intensity and the duration of the clonic phase (*r* = −0.349, *p* < 0.05), but not that of the tonic phase (*r* = −0.171, *p* = 0.266) (Figure 4).

**Table 1:** **Mean (SD) age, stimulus intensity, seizure duration, tonic phase, and clonic phase**.

Mean (SD)	Male (*n* = 19)	Female (*n* = 25)
Age (year)	39.68 (8.9)	37.8 (9.32)
Stimulus intensity (J)	28.73 (6.28)	28.87 (6.58)
Seizure duration (s)	26.66 (5.4)	28.87 (6.83)
Tonic phase (s)	8.9 (1.5)	9.36 (1.35)
Clonic phase (s)	17.76 (4.71)	19.51 (6.04)

**Table 2:** **Correlation of stimulus intensity with seizure duration, tonic phase, clonic phase, and age**.

	Age			Stimulus intensity		
	Seizure duration	The tonic phase	The clonic phase	Seizure duration	The tonic phase	The clonic phase
*r*	−0.435	−0.158	−0.455	−0.345	−0.171	−0.349
*P* value	0.003**	0.306	0.002**	0.022*	0.266	0.02*

*Correlation is significant at the 0.05 level (two-tailed).

**Correlation is significant at the 0.01 level (two-tailed).

## Discussion

Over the past decade, traditional practical ECT theory has considered the therapeutic effect of ECT input-current duration to be correlated to age, gender, and seizure duration. Therefore, to successfully affect patient cure, the input-current intensity and duration were modulated according to these parameters in the textbook and clinical practical manual. For instance, elderly patients may require longer duration and intensity of electro-input to achieve a seizure state of sufficient duration. Furthermore, in clinical practice, the prediction of the therapeutic effect of ECT mostly depends on whether the induced seizure duration exceeds 15 seconds. If the seizure-duration shorter than 15 seconds, the therapeutic trial is generally defined as a fail trial, and the patient accepts another ECT trial following parameter modulation and clinical assessment. Seizure duration thus becomes a substitute criterion for medical decisions regarding ECT and for developing an index of therapeutic effect.

The present data suggest that there is a negative correlation between seizure duration and age. According to the anticonvulsant hypothesis, the anticonvulsant effect could suppress the association with age. This result is congruent with previous studies (4–6). Moreover, our findings indicate that there is a negative correlation between the clonic phase of seizure and age, but not the tonic phase. Additionally, there is a negative relation between the clonic phase of seizure and stimulus intensity, but not the tonic phase. The two-correlation data may not be an isolated observation as age may represent an index of brain degeneration and neuronal density (7, 8), in which case the input intensity of the electric current (stimulus) will be negatively correlated with age.

### Age vs. seizure duration

At present, there are few studies that explore the effect of the clonic phase in ECT. This is a preliminary study to assess the relationship between seizure duration during the ECT and subject characteristics. We cannot measure brain activity directly during the ECT; however, we can collect the information from the patients’ responses after the ECT. Previous ECT-related studies (9) have suggested that there is a relationship between seizure duration and age. For example, previous studies (9) have suggested that seizure duration was shorter in older patients. The present study supports not only the previous finding between seizure duration and age but also the relationship between seizure duration and stimulus intensity (10).

### Age vs. intensity

This study found that there was no correlation between gender and seizure duration or stimulus intensity. These findings are consistent with the previous literature on ECT (7). Some studies suggest that gender differences can be determined in the seizure threshold (9). Therefore, the gender difference may be observed when the internal factors of patients are controlled for. It remains unclear how ECT produces a temporary therapeutic effect on schizophrenic and major depression patients. We first investigated the relationship between the clonic phase and the patient variables. It seems to imply the clonic phase may play an important role during ECT treatments. The clonic phase may represent the self-brain regulating activities after an external current input. The relationship between clonic activities and clinical efficacy could be the subject of future research.

### Seizure duration vs. intensity

In sum, the findings of the present study indicate that a relationship exists between the clonic phase of seizure and patient age, but not between the tonic phase and patient age. Furthermore, we also found a correlation between the clonic phase and stimulus intensity, but not between the tonic phase and stimulus intensity. We speculate that the anticonvulsant effect is associated with age and the clonic phase during ECT. Our study predominantly concerned the association between the clonic phase of a seizure and age with respect to the anticonvulsant hypothesis. Additional studies are necessary to clarify the details of brain activation during the clonic and tonic phases (11).

### Limitations

Notably, the present post-analysis research possessed some general limitations of ECT retrospective chart survey. The first limitation of this study is due to the study is a retrospective research. It is difficult to recollect the detail information of some patient’s treatment-history. Further, this study is also limited to the sample size. However, this research still presented a significant finding.

## Conclusion

In short, the findings of this study indicated that the therapeutic effect was not associated with the tonic phase, but was precisely correlated with the duration of the clonic phase. Namely, the clonic phase may be more critical as a clinical index of ECT assessment and ECT therapeutic effect. The observations presented here implied that the present ECT paradigm in clinical practice could require refinement. On the other hand, significant sample-size re-evaluation may be needed for advanced verification.

## Author Contributions

Chao-Chih Wang and Chih-Chieh Tseng have made the contributions to thought, data collection, and interpretation as well as drafting preliminary manuscript. Ching-Hung Lin and Yao-Chu Chiu participated in consulting of data analysis, provided some discussion, and refining the manuscript. All authors gave final approval of the version to be published.

## Conflict of Interest Statement

The authors declare that the research was conducted in the absence of any commercial or financial relationships that could be construed as a potential conflict of interest.
